# Assisted reproduction technology and reproductive landscapes in a global era

**DOI:** 10.5935/1518-0557.20190072

**Published:** 2020

**Authors:** Marilena C. D. V. Corrêa

**Affiliations:** 1 Senior Associate Professor at the Social Medicine Institute of the State University of Rio de Janeiro, Brazil; 2 Visiting Researcher at CERMES3, France

This editorial aims to present a recent scientific publication on the theme of cross-border reproduction, authored by French sociologist and demographer Virgine Rozée (2018). Her rigorous innovative work in the social area, supported by empirical studies (conducted in India, Bolivia, European countries, among others), gives visibility to the challenges introduced by the biomedicalization of human reproduction in times of globalization. In a setting of transnationalization, services, techniques, practices, isolated human reproductive elements (eggs, frozen embryos), people (professionals, relatives, donors, surrogate mothers), reproductive projects, money and other elements circulate unreservedly among countries and diverse regions of the world.

As I proposed in a very different context, in a research project conducted in the area of *social studies of innovation and techniques, in vitro* embryos may be regarded as the most finished form of what has been described as "socio-technical hybrids." As a result of the advent of reproductive material conservation techniques, frozen embryos have become stockable and thus further expanded the range of possibilities for individual or couple reproductive projects. As socio-technical hybrids, embryos expanded the borders of knowledge and technology, intertwining science, biomedical services, the pharmaceutical and equipment industry, public policy, health regulations and legislation, and professional ethics. However, the movement toward this complex array of *technical* instances had its onset in unmet socially and individually - patients and users - constructed needs to start a family or reproduce.

The use of assisted reproduction technologies (ART) has raised fundamental questions around the dominant social norms about gender, reproduction, and choice in the reproductive arena. Even though they vary over time, across countries and cultures, norms necessarily imbricate biological and social dimensions (Loyola, 1992): who has a legitimate claim over having children or forming a family? What about the urgency of permanence via procreation? If, on the one hand, the answers to these and other questions still follow social representations and attitudes predominantly oriented by heteronormativity, on the other hand, changes in the plane of individual choice become increasingly evident and further challenge the core of social norm, in addition to calling for better answers around the application of possible biomedical technologies and the development of innovative legal solutions.

Rozée (2018) offers insight into these changes and invites readers to situate themselves in what she defined as the "*margins*" - places that might encourage new perspectives on the project of having children and drive the formation of a wide variety of family arrangements. According to the author, the *margins* also allow one to see the paths taken by men and women, in the form of the actions and attitudes adopted by each individual, as they search for family and parenting projects that might (or not) rely on third parties (physically, genetically, biologically) for the production of babies.

While reproductive biological elements cross borders and may be geographically scattered, available for reassembly according to the needs of parents-to-be, *reproductive work* remains much more closely linked to the female body. In the transnational reproductive chain, reproduction remains a gendered work. This is clear when one looks for egg donors in a cross-border dimension and even more when surrogacy is needed. Undoubtedly, the biomedicalization of reproduction by ART has contributed greatly to the deconstruction of gender-related traditional reproductive social norms. But reproductive medicine has yet to confront the issues regarding the limits of these deconstructions in relation to different risks for men and women, considering that the female body is responsible for much of the reproductive work in accessing a family (Tain, 2013).

Marcia Inhorn (2018) conducted seminal socio-anthropological studies known for the quality and diversity of researched contexts. In reference to the Muslim world, she author coined the expression *reproscapes* - "reproductive landscapes" - to allude to the vicissitudes of the cross-border path that exacerbates focus on cultural barriers, regulatory norms, development of different professional ethics across countries, and economic issues alike.

In fact, it is worth reflecting on the permanence of problems linked to socioeconomic inequalities and the exercise of individual autonomy. Reproductive tourism, an expression of negative connotation, is most commonly used to refer to cross-border arrangements involving people from very economically unequal centers - high *vs.* low income countries - and/or when compensation for the use (or sales) of eggs and surrogacy are made possible or facilitated. Beyond the economic entanglements, these have always been aspects of strong inquiry regarding freedom of choice and the exercise of autonomy.

Rozée's work (2018), in my understanding, brings a unique contribution to the answers and discussions held on these matters with the adoption of the idea of *resilience*. Recognizing that imbalances and inequalities may bring "risks" (in a broad sense) to negotiations of transnational reproductive arrangements, resilience entails a work of resistance to norms, especially on the part of women - resistance to marital heteronormativity, to legal or cultural interdictions, justified as a protective measure against the exploitation of women. Seen from the *margins*, what was being mechanically analyzed as vulnerability might reveal other realities of transgression and change.

Unfortunately, there is not enough room here to discuss the rich details the author presents in her thesis. But from the *margins*, the exercise of resistance and autonomy by stakeholders reveals a transformed vision of the vulnerability of women located in less developed countries and their sociodemographic profiles. In low-income communities, women are neither the poorest nor the least educated, thus indicating that these are reflected, not survival life strategies. The author cites interesting examples, such as the case of South Africa, where most donors were white, middle-class university students who sought to "finance a professional career" in exchange for their services; or in India, where women with the same profile aimed to fund "children's studies". Moreover, the practice of transnational reproduction occurs even in Europe, due to differences of legal and bioethical restrictions among countries. Perhaps the reproductive transit in this region is the densest, given the cultural similarities; but the legal barriers and/or the public allocation of health resources also play a central role in such transit.

From this point of view, it is possible to see, in the global chain of reproductive work, different dynamics and possibilities, including the transformation of vulnerable "mothers and wives" into resilient women who turn reproduction into a place of power.
